# Breaking Through Resistance: Carbapenem-Resistant Nocardia?!—A Case Report

**DOI:** 10.3390/medicina60111863

**Published:** 2024-11-14

**Authors:** Vlad-Sabin Ivan, Paul-Gabriel Ciubotaru, Liviu Cirin, Florin Giru, Dan Costachescu, Daniel-Florin Lighezan, Roxana Buzas

**Affiliations:** 1 Department V, Internal Medicine I, Center for Advanced Research in Cardiovascular Pathology and Haemostaseology, “Victor Babeș” University of Medicine and Pharmacy Timișoara, 300041 Timisoara, Romania; ivan.vlad@umft.ro (V.-S.I.); cirin.liviu@umft.ro (L.C.); dlighezan@umft.ro (D.-F.L.); buzas.dana@umft.ro (R.B.); 2 Institute of Cardiovascular Diseases Timisoara, 300310 Timisoara, Romania; florin.giru@umft.ro; 3 Orthopedics-Traumatology, Urology, Radiology and Medical Imaging Department, “Victor Babeș” University of Medicine and Pharmacy Timișoara, 300041 Timisoara, Romania; costachescu.dan@umft.ro

**Keywords:** *Nocardia*, carbapenems, infection

## Abstract

*Nocardia* is an emerging bacterial disease that often affects patients with compromised immune systems. As the number of patients undergoing solid organ transplants continues to rise, and as more cancer survivors are treated with long-term immune-modifying agents, corticosteroids, and immunosuppressive medications, organisms that typically pose no harm are becoming a public health concern. Carbapenems are usually a second-line therapy in the setting of *Nocardia* infections. We present the case of a patient who had an immunocompromised status and was diagnosed with *Nocardia farcinica*, which showed in vitro resistance to carbapenems. The symptoms were non-specific and had a common presentation with headache, fatigue, and a nonresponsive cough to usual cough drugs, although the infection was disseminated and had severe CNS and ocular involvement. The clinical course worsened when the carbapenem was withdrawn and markedly improved when it was re-administered, although the initial antibiogram showed resistance to carbapenems. Despite the observed in vitro resistance in the laboratory, the re-administration of carbapenems was beneficial, as both inflammatory markers and clinical status showed improvement. This was based on clinical judgment, which carefully evaluated the associated risks and benefits. Also, this raises the question of potential risks to develop real resistance to carbapenems of some *Nocardia* species, which could pose a significant challenge to healthcare. It could become a serious healthcare problem in the future and should prompt active environmental testing.

## 1. Introduction

In immunocompromised patients, the risk of opportunistic infections is high. Viral, bacterial, or fungal agents contribute to increased morbidity and mortality [1].

These patients have an incompetent immune system either as a primary defect of the innate immune system or adaptive immunity, immune dysregulation [2], or secondary to immunosuppressive therapy and other prior therapies (chemotherapy and longtime administration of steroids or antimicrobials), but also in the case of organ transplant, altered integrity of the mucocutaneous barrier and metabolic and endocrine disorders.

Nocardiosis is an infectious disease caused by *Nocardia*, which is a Gram-positive bacillus with branching hyphae found throughout the environment in soil, decomposing vegetation, and other organic matter [3], that can cause localized or disseminated disease. It usually manifests as localized cutaneous and pulmonary disease with abscess formation. The disseminated nocardiosis can affect any organ with lesions in the brain with the meninges being the most common [4].

There were 54 known *Nocardia* species, with *N. nova* complex, *N. abscessus* complex, *N. transvalensis* complex, and *N. farcinica* being the most common species involved in human pathology, but because of recent genetic developments, there are over 100 species described [5]. There are approximately 500–1000 cases per year in the United States, and Italy reported around 100 cases during a five-year period in Europe [3].

In the last two decades, the incidence rate of Nocardiosis increased from 0.33 to 0.87 per 100,000 patients, in certain regions such as North America [6]. This rise can be attributed to various factors such as aging, increased use of immunosuppressive drugs, chemotherapy, biological agents, and monoclonal antibodies. All of these factors impact the ability of the host to adapt and react to bacterial infections.

## 2. Case Report

A male patient, employed in the forestry industry, aged 58 and a nonsmoker, was diagnosed with chronic membranous glomerulonephritis and nephrotic syndrome in 2016. He was given 150 mg of azathioprine (Imuran^®^) once a day (2 mg/kg) for immunosuppressive therapy. However, there was no improvement in proteinuria reduction, so after 4 weeks, methylprednisolone, an oral corticoid, was added to his treatment (64 mg once a day). The steroid treatment helped to reduce proteinuria to about 500 mg/L and it remained stable. The azathioprine treatment was stopped after 8 weeks because the patient developed an extensive area of herpes zoster covering 3 intercostal dermatomes. The proteinuria was dose-dependent, and at lower doses than the 16 mg methylprednisolone threshold, the proteinuria was not controlled.

The patient continued to receive chronic steroid treatment and had regular check-up visits in the nephrology department between 2016 and 2022. He was admitted several times for edema during this period and also developed hypertension and steroid-induced diabetes mellitus. To manage these conditions, he was prescribed ACE inhibitors, calcium channel blockers, high-potency statins, and insulin in a basal-bolus regimen.

During this period, the serum creatinine remained at a stable level of 1 mg/dL.

**Figure 1:**
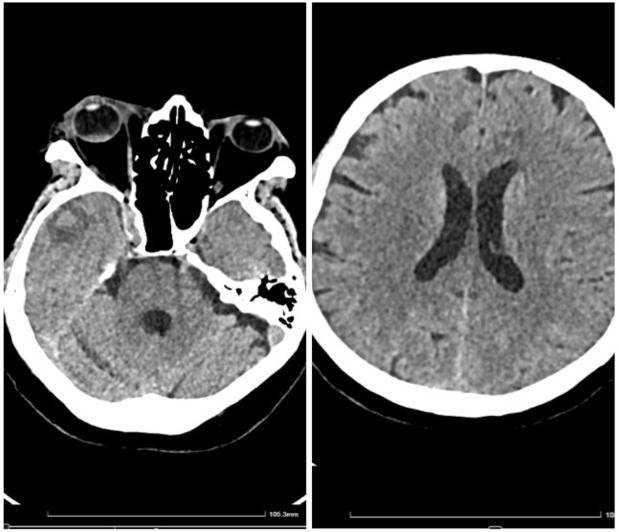
Initial cranial CT (no contrast).

In early May 2023, the patient developed a respiratory illness that was treated with iv gentamycin and ceftriaxone for 7 days in a territorial hospital and was discharged. In late May 2023, he developed right retrorbital pain, with a painful right red eye with acute vision loss, headaches, 7 kg weight loss, and aggravated fatigue that were not controlled with usual medication and cough. The patient was admitted to the Internal Medicine Department for further evaluation. We performed a native cranial CT (Figure 1) and an MRI without contrast which showed multiple lesions—10—that were classified as undetermined lesions on the CT scan and possible abscessed metastases on the MRI.

**Figure 2:**
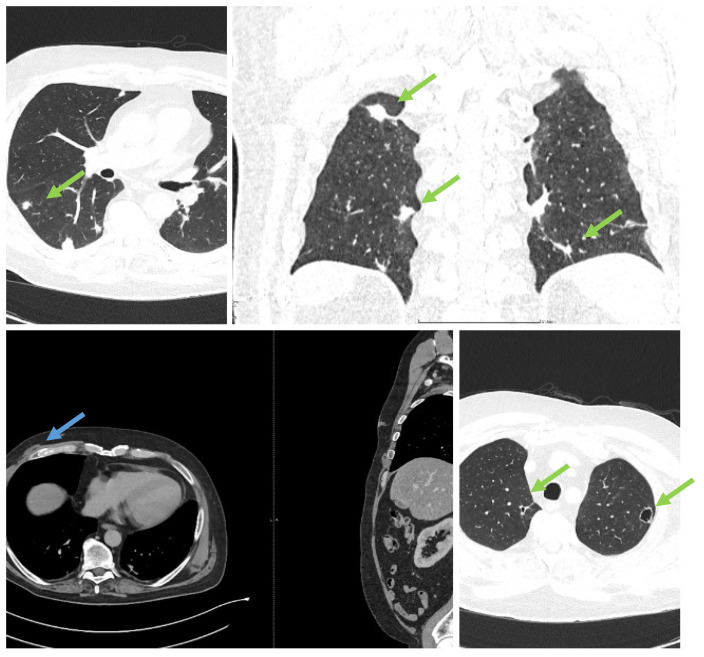
CT with contrast, showing disseminated lesions in the lungs, abdomen, and soft tissues (the green arrows indicate disseminated abscesses, blue arrow indicates drained abscess).

**Figure 3:**
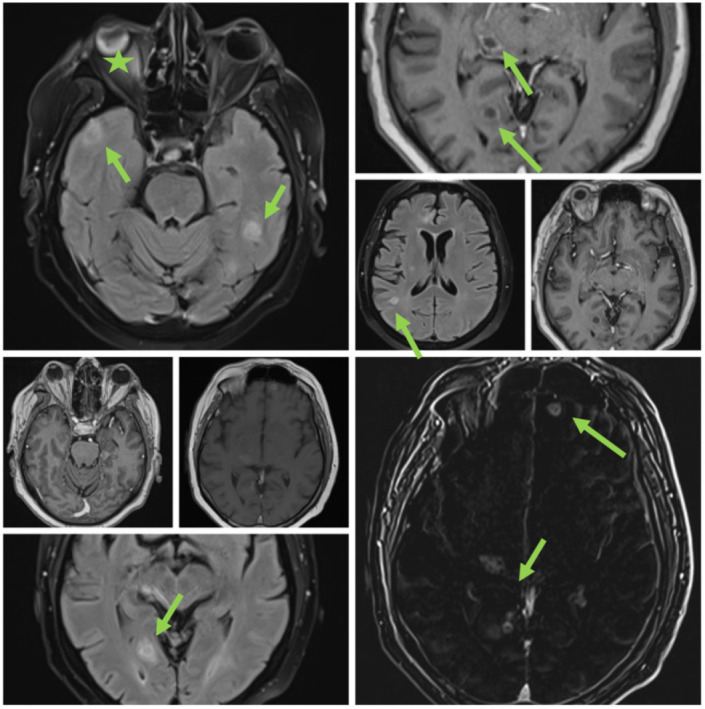
MRI. (Ring-enhancing lesions are present in all cerebral lobes and the thalamus. Different sequences showing brain abscesses are indicated by green arrows; the star indicates eye involvement).

It was suspected that there was a metastatic tumor, and a full-body CT scan was performed (Figure 2). The CT scan discovered multiple abscesses in the lungs and under the skin. A second contrast MRI (Figure 3) scan was conducted which revealed abscesses in all parts of the brain, including the right thalamus and left cerebellar hemisphere, with ring-like contrast-enhancing lesions. However, the lesions were relatively stable compared to the previous MRI.

The full-body CT scan showed multiple nodular cavitating lesions spread throughout the pulmonary area and several subcutaneous abscesses in various locations, with one notable abscess in the distal portion of the anterior serratus muscle and right pre-pectoral subcutaneous fat. Additionally, there were multiple nodules on the peritoneum and perisplenic fat, all measuring between 2 and 3 cm in diameter.

**Figure 4:**
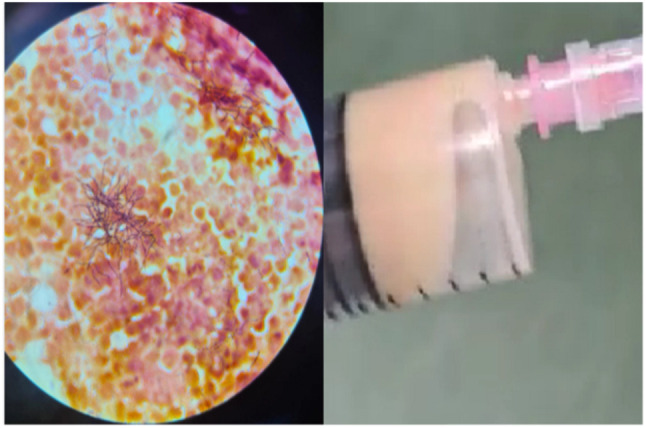
Macroscopic aspect of the evacuated subcutaneous abscess (**right**) and microscopic aspect with ramified filamentous Gram-positive bacilli growing in an aerobic environment (**left**).

The initial bloodwork showed mild leucocytosis (15,000/mm^3^), with normal procalcitonin and a CRP of 11.6 mg/L, an ESR of 49 mm/h, a 2.3 g proteinuria, a creatinine level of 1.2 mg/dL, and otherwise unremarkable biochemistry. Blood cultures were sterile, and a subcutaneous abscess was drained and sent to the laboratory for analysis (Figure 4). The patient was started on empirical antibiotics with iv meropenem 500 mg t.i.d. and amoxicillin/clavulanate 1 g b.i.d. The initial clinical course was favorable, with remission of head pain.

The microbiological work-up and antibiogram showed that the species was in fact rod-shaped with filamentous ramification Gram-positive bacteria, which was identified as *Nocardia farcinica* which had a susceptibility to linezolid and was resistant to imipenem, amikacin, and intermediate to amoxicillin/clavulanate (Figure 4). The patient was started on a combination of linezolid 600 mg b.i.d., and trimethoprim/sulfamethoxazole 400 mg/80 mg t.i.d. Also, a meropenem resistance study was performed which showed resistance to meropenem, and thus the study was stopped on day 6 and continued on the other two antimicrobials, but relapse occurred, with the patient reporting a 10 on the Borg pain scale (Table 1).

**Figure 5:**
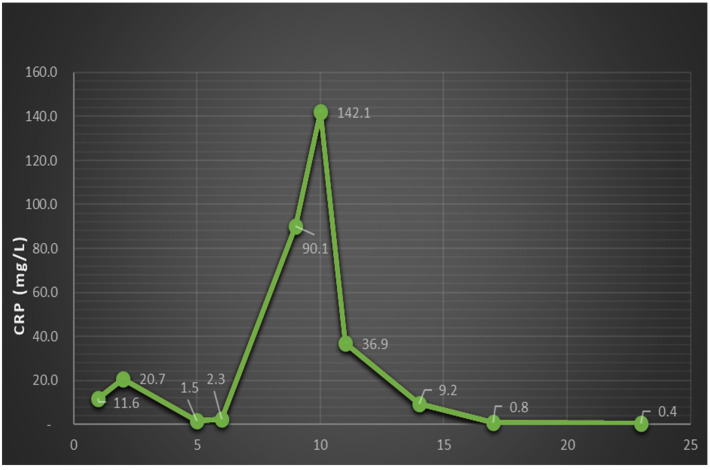
CRP level dynamics (the spike indicates meropenem withdrawal and corroborates with the sharp increase in headache intensity and retroorbital pain).

The subsequent clinical course after meropenem was stopped when it was relatively severe, with the patient developing intense right eye pain and a sudden drop in visual acuity, which required a step-up of pain medication; eventually, the patient received a combination of metamizole, tramadol, acetaminophen, pethidine, and nefopam. Even so, the patient declared a 10/10 Borg RPE pain score. The second MRI showed right posterior eye pole thickening and retroorbital edema, highly suspicious retinal detachment, which the ophthalmology consult confirmed as no light perception, so the decision was made to re-administer meropenem, again with a decrease in the pain scale. Also, there was a spike in CRP level that corroborated with the withdrawal of meropenem and improvement with its re-administration (Figure 5). After re-administration of meropenem, the clinical course dramatically improved, and the patient received only on-demand acetaminophen.

The patient also received a regimen aimed at reducing brain edema with dexamethasone, mannitol, and complex B vitamin, altogether reducing the proteinuria to 0.3 g/L.

The total duration of the three antibiotics combined was 35 days, and the patient was discharged with a diagnosis of disseminated *Nocardia farcinica* infection, recommending a continuation of trimethoprim/sulfamethoxazole for at least one year.

**Figure 6:**
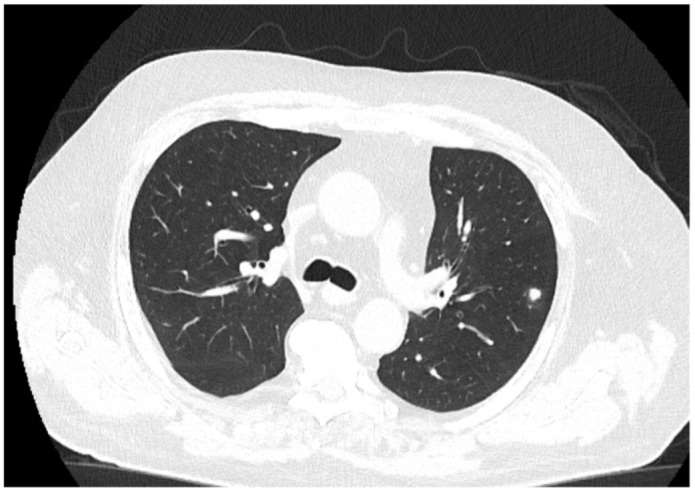
Follow-up Ct at three months. The chest CT does not show any cavitating lesions, with residual scarring and healing processes of the lesions.

The patient received a follow-up brain MRI and chest CT (Figure 6) at 3 months, which showed complete remission of the brain abscess.

## 3. Discussion

Although nocardiosis is a predominantly tropical disease, *Nocardia* spp. are found all over the world, with some species found more commonly in specific areas [7,8]. There are over 100 species described; for example, *Nocardia brasiliensis* is found more often in tropical regions, and *Nocardia nova* is found predominantly in northern states and the Midwest of the USA, while in southeastern states, *Nocardia farcinica* is more common [9]. In Europe, in France, *Nocardia farcinica* and *Nocardia abscessus* were found in one-fifth of the cases each [10].

*Nocardia* can be responsible for severe opportunistic infections in immunocompromised patients and patients with chronic bronchopulmonary diseases.

Recipients of solid organ transplants and individuals with HIV are usually the most common type of patients because of their ineffective immune systems [9].

Pulmonary nocardiosis is the most common form of the disease, often mimicking tuberculosis or other fungal infections. Some estimates suggest that more than two-thirds of the patients have only pulmonary involvement [11]. Cutaneous involvement along lymphadenopathy is also frequently described [3]. The disseminated form involves at least three different organs, with virtually any organ involved. CNS involvement usually means a disseminated form, with patients describing paresthesia, numbness, motor function loss, or focal neurological signs.

This case that we present has some peculiarities. At first, although the patient had some exposure because of working in forestry, he did not mention any cutaneous lesion in the preceding months, which could provide an entry point, and we suspect that the patient’s initial form of exposure was pulmonary through inhalation. Secondly, the patient did not have any major neurologic symptoms as expected, although the retroorbital pain grew stronger, which required high doses of several strong antalgic drugs. Thirdly, the meropenem treatment improved the clinical outcome even if the antibiogram showed resistance, which could mean that it probably had a synergistic effect due to the linezolid–trimethoprim/sulfamethoxazole combination.

The patient is an immunocompromised one, because of the long-term use of his cortico-sensitive glomerulonephritis. Indeed, after high doses of dexamethasone were administered for the brain abscess, the proteinuria dropped to 0.4 g/24 h, confirming the cortico-sensitivity of the glomerulonephritis.

The presentation in this case with cavitating lesions is highly suspicious in its disseminated form in immunocompromised patients [12] and is associated with high doses of corticosteroids. Steinbrink et al. [12] found 135 patients in whom cavitation occurred only in the immunocompromised patients, with almost 20% mortality. The lung was the most common site of infection, and only 27% had a disseminated form, with *Nocardia asteroides* being the most common in the immunocompromised patients (67%), and *Nocardia farcinica* being present in only 16% of the cases of the same group.

Whether dissemination ensues following respiratory tract infection is dependent on the containment of the initial infection by the host. With obvious immunosuppression, such as with high-dose corticosteroids, dissemination is more common. In our patient, cutaneous and subcutaneous infections could have been the first point of entry as the patient could have been easily bruised at work, but it is described as more common in *Nocardia brasiliensis* infections [13].

How our patient was infected is only hypothesized as there was no evident point of entry. Other authors also have not found a point of entry, especially for *Nocardia beijingensis*, which is particularly of interest because, since its discovery in 2001, there have been ten cases reported in immunocompetent patients. One case was reported in a worker in the landfill industry [14].

Cerebral involvement accounts for 44% of cases when the disseminated form occurs, and when a pulmonary diagnosis of *Nocardia* is established, and a cerebral mass is found, a brain biopsy is not needed [15]. Although the standard regimen is trimethoprim/sulfamethoxazole in a simple pulmonary form, the disseminated form requires multiple antibiotics in an intravenous formulation for extended periods of time, especially in an immunocompromised host [16].

Mimicking metastatic disease from different cancers in the lungs or brain is common, but this type of infection is often overlooked, and the focus is placed on diagnosing the suspected neoplastic disease. With non-specific presentations and an insidious nature, diagnosing *Nocardia* based on clinical findings and usual laboratory data is difficult and time-consuming [17].

Regarding the patients’ laboratory findings, there are several interesting issues to note. Leucocytosis is present, but it can be misleading because chronic corticosteroid therapy increases the white blood cell count. Additionally, corticosteroid therapy reduces inflammation, which can mask acute phase reactants [18]. The CRP levels at admission are not significantly elevated and show a quick improvement following initial antibiotic treatment. However, there is a spike in CRP that correlates with the withdrawal and subsequent re-administration of meropenem. This suggests an in vivo susceptibility, which contradicts the in vitro results. Some studies have indicated that there are no differences in C-reactive protein (CRP) levels between patients with nocardiosis and those with pneumonia, suggesting that CRP cannot effectively distinguish the severity of the disease [19]. However, some case reports have shown elevated CRP levels in patients with nephrotic syndrome and nocardiosis, with levels reaching around 100 mg/L [20]. A recent study from Taiwan [21] indicated that the susceptibility to ceftriaxone, imipenem, and amoxicillin/clavulanate decreases over time, while it remains stable for trimethoprim/sulfamethoxazole and linezolid. The authors emphasize the importance of identifying each bacterial species to determine the appropriate antibiotic regimen, as different species may exhibit varying susceptibilities. However, for disseminated infections, combination therapy should be initiated.

The most interesting aspect is the response to meropenem, even though the antibiogram showed resistance to this drug, highlighting a possible potentiating effect of one on another. We also presumed that it might have been other bacteria that we did not identify, and that could have been the cause of the orbital pain and subsequent spike in CRP levels, but we cannot ascertain this for sure because retroorbital pain can be caused by *Nocardia* endophthalmitis.

## 4. Conclusions

We consider this case an interesting one, as the resistance to trimethoprim/sulfamethoxazole is more common, and in the case of carbapenem resistance, at least in vitro, this should warrant closer monitoring of this often non-diagnosed infectious disease in a population that is high-risk and has frailty.

The clinical course was positive, and we believe that evaluating the patient holistically while weighing the risks and benefits in each case can yield favorable outcomes. Interdisciplinary management is crucial in these complex cases as results from different investigations can sometimes be conflicting or unexpected, and should warrant careful nursing.

## Figures and Tables

**Table 1:** Antibiogram for *Nocardia farcinica* (R—resistant; I—intermediate resistance; S—sensitive; Value MIC—value of minimal inhibitory concentration).

Antibiotic	Value MIC	U.M.	Characteristic
Amikacin	2	μg/mL	R
Linezolid	0.75	μg/mL	S
Imipenem	>32	μg/mL	R
Amoxicillin/Clavulanic Acid	8	μg/mL	I
Meropenem	>32	μg/mL	R

## Data Availability

Data will be made available on valid written requests to the corresponding authors.

## References

[1] Fishman J.A. (2013). Opportunistic infections—Coming to the limits of immunosuppression?. Cold Spring Harb. Perspect Med..

[2] Riccardi N., Rotulo G.A., Castagnola E. (2019). Definition of Opportunistic Infections in Immunocompromised Children on the Basis of Etiologies and Clinical Features: A Summary for Practical Purposes. Curr. Pediatr. Rev..

[3] Rawat D., Rajasurya V., Chakraborty R.K., Sharma S. (2024). Nocardiosis. StatPearls [Internet].

[4] Wilson J.W. (2012). Nocardiosis: Updates and clinical overview. Mayo Clin. Proc..

[5] Brown-Elliott B.A., Brown J.M., Conville P.S., Wallace R.J. (2006). Clinical and laboratory features of the Nocardia spp. based on current molecular taxonomy. Clin. Microbiol Rev..

[6] Tremblay J., Thibert L., Alarie I., Valiquette L., Pépin J. (2011). Nocardiosis in Quebec, Canada, 1988–2008. Clin. Microbiol. Infect..

[7] Farina C., Boiron P., Ferrari I., Provost F., Goglio A. (2001). Report of human nocardiosis in Italy between 1993 and 1997. Eur. J. Epidemiol..

[8] Duggal S.D., Chugh T.D. (2020). Nocardiosis: A Neglected Disease. Med. Princ. Pract..

[9] Gupta S., Grant L.M., Powers H.R., Kimes K.E., Hamdi A., Butterfield R.J., Gea-Banacloche J., Vijayvargiya P., Hata D.J., Meza Villegas D.M. (2023). Invasive Nocardia Infections across Distinct Geographic Regions, United States. Emerg. Infect Dis..

[10] Lebeaux D., Bergeron E., Berthet J., Djadi-Prat J., Mouniée D., Boiron P., Lortholary O., Rodriguez-Nava V. (2019). Antibiotic susceptibility testing and species identification of Nocardia isolates: A retrospective analysis of data from a French expert laboratory, 2010-2015. Clin. Microbiol. Infect..

[11] Vohra P., Sharma M., Yadav A., Chaudhary U. (2013). Nocardiosis: A review of clinico-microbiological features. Int. J. Life ScBt. Pharm. Res..

[12] Steinbrink J., Leavens J., Kauffman C.A., Miceli M.H. (2018). Manifestations and outcomes of nocardia infections: Comparison of immunocompromised and nonimmunocompromised adult patients. Medicine.

[13] Parvu M., Schleiter G., Stratidis J.G. (2012). Skin infections caused by Nocardia species. Infect. Dis. Clin. Pract..

[14] Lam J.C., Chan W.W., Walsh J.F. (2022). Disseminated nocardiosis in an immunocompetent host with occupational exposure. IDCases.

[15] Sorrell T.C., Mitchell D.H., Iredell J.R., Mandell G.L., Bennett J.E., Dolin R. (2005). Nocardia species. Principles and Practice of Infectious Diseases.

[16] Arjun R., Padmanabhan A., Reddy Attunuru B.P., Gupta P. (2016). Disseminated nocardiosis masquerading as metastatic malignancy. Lung India.

[17] Walter T., Zadeh G., Hawryluk G., Krzyzanowska M.K. (2013). Nocardia brain abscess in a patient treated with everolimus for a metastatic insulinoma. J. Clin. Oncol..

[18] Mank V., Azhar W., Brown K. (2024). Leukocytosis. StatPearls [Internet].

[19] Margalit I., Goldberg E., Ben Ari Y., Ben-Zvi H., Shostak Y., Krause I., Muhsen K. (2020). Clinical correlates of nocardiosis. Sci. Rep..

[20] Cheng Y., Wang T.-Y., Yuan H.-L., Li W., Shen J.-P., He Z.-X., Chen J., Gao J.-Y., Wang F.-K., Gu J. (2022). *Nocardia* Infection in Nephrotic Syndrome Patients: Three Case Studies and A Systematic Literature Review. Front. Cell. Infect. Microbiol..

[21] Lao C.K., Tseng M.C., Chiu C.H., Chen N.Y., Chen C.H., Chung W.H., Liu T.P., Lu J.J., Lai H.C., Yang L.Y. (2022). Clinical manifestations and antimicrobial susceptibility of Nocardia species at a tertiary hospital in Taiwan, 2011–2020. J. Formos Med. Assoc..

